# Transactions of California State Homœopathic Society

**Published:** 1891-01

**Authors:** 


**Transactions of the Fourteenth Annual Session of the California State Homœopathic Society.** Held at San Francisco, Cal., May 14th, 15th, 1890. Vol. I.

Our California brethren have succeeded in giving us in the above volume a unique work. It contains more genuine Homœopathy than any recent volume of a similar character that we have seen. Hahnemann and his work seem to be well known in California. The Bureau of Materia Medica deserves especial notice. Its chairman, Dr. A. McNeil, has succeeded in having excellent work done. We trust that we may be able to greet future volumes from this society with the heartiness which we give this. We advise our readers to apply for a copy to Dr. Geo. H. Martin, San Francisco, Cal.

